# Sex differences in the association between obesity and albuminuria among Korean adults: a cross-sectional study using the Korea National Health and Nutrition Examination Survey data

**DOI:** 10.1007/s10157-016-1238-z

**Published:** 2016-02-22

**Authors:** Hye-Mi Noh, Un-Young Kim, Yong Soon Park, Young Rim Song, Hye-Young Oh, Kyung-Hee Park, Yu-Jin Paek, Yong Kyun Roh, Hong Ji Song

**Affiliations:** 1 Department of Family Medicine, Hallym University Sacred Heart Hospital, Anyang, Gyeonggi-do 431-796 Republic of Korea; 2 Daejeon Woori Spine Hospital, Daejeon, Republic of Korea; 3 Department of Family Medicine, Chuncheon Sacred Heart Hospital, Hallym University College of Medicine, Chuncheon, Republic of Korea; 4 Department of Internal Medicine, Kidney Research Institute, Hallym University Sacred Heart Hospital, Anyang, Gyeonggi-do Republic of Korea; 5 Department of Family Medicine, Kangnam Sacred Heart Hospital, Seoul, Republic of Korea

**Keywords:** Albuminuria, Obesity, Waist circumference (WC), Body mass index (BMI), Waist-to-height ratio (WHR)

## Abstract

**Background:**

The association between obesity and albuminuria in the general population remains unclear. We aimed to identify the association between obesity and albuminuria as well as sex differences regarding the associations using several obesity indices, including waist circumference (WC), body mass index (BMI), and waist-to-height ratio (WHR).

**Methods:**

This study included 3841 subjects (1730 males and 2111 females; age 20–80 years) who participated in the Fifth Korea National Health and Nutrition Examination Survey conducted in 2011. Subjects with hypertension, diabetes, renal failure, or a malignant tumor and those who were pregnant or menstruating were excluded. Albuminuria was defined as a urinary albumin-to-creatinine ratio ≥30 mg/g. Anthropometric parameters were categorized into sex-specific quartiles. Logistic regression models were used to assess the associations between each anthropometric parameter and albuminuria.

**Results:**

All of the obesity indices of the fourth quartile group of females showed a twofold higher risk for albuminuria than the second quartile group, and it was persistently significant after adjusting for age, smoking, and physical activity. After further adjustment for high blood pressure and impaired fasting glucose and triglyceride levels, WC and BMI of the fourth quartile group of females still showed a significantly higher risk for albuminuria than the second quartile group (odds ratios 1.96 and 2.24; 95 % confidence intervals 1.03–3.74 and 1.15–4.37). None of the associations between albuminuria and the obesity indices were significant in males.

**Conclusion:**

Higher WC and BMI were significantly associated with the risk of albuminuria among females, but not males.

**Electronic supplementary material:**

The online version of this article (doi:10.1007/s10157-016-1238-z) contains supplementary material, which is available to authorized users.

## Introduction

Obesity is a major risk factor for cardiovascular disease, diabetes mellitus, and hypertension [1]. The prevalence of obesity has increased worldwide. According to Korea Health Statistics 2011, obesity affects one-third of Korean adults [2]. Chronic kidney disease (CKD) is also a global health concern with increasing incidence and prevalence [3]. Traditional risk factors for CKD include diabetes mellitus, hypertension, and metabolic syndrome [4], which are health problems related to obesity. Microalbuminuria is an early marker of kidney injury and a predictor of end stage-renal disease (ESRD) and cardiovascular mortality [5, 6].

Previous studies have suggested that obesity increases the risk of CKD and albuminuria; however, results have been inconsistent due to the obesity indices used, confounding factors, and the sex ratio of the population. A longitudinal cohort study from the Framingham Offspring Study reported that higher body mass index (BMI) is related to developing CKD [7]. A retrospective case–control study showed that obesity, as defined by BMI among both males and females, is linked to an increased risk for CKD [8]. Another study found an association between higher BMI and the risk for ESRD in both sexes [9]. However, studies from Singapore and Japan have suggested a sex-specific association between BMI and CKD [10–12]. They reported a positive correlation between higher BMI and CKD among males, but not females. In contrast, Chinese studies have reported that abdominal obesity is related to CKD or albuminuria among females, but not males. Lin et al. found that the waist-to-height ratio (WHR) is associated with CKD in Chinese females, whereas the association was not significant in males [13]. Furthermore, another Chinese study showed that central obesity, as measured by waist circumference (WC), indicates a tendency to develop albuminuria independently of BMI. These findings were most pronounced in females [14]. Although two other Korean studies demonstrated that obesity is associated with albuminuria among the general population and hypertensive adults [15, 16], they did not consider sex differences between obesity and albuminuria.

These controversies about obesity and renal dysfunction may be due to differences in ethnicity, sex, obesity indices, or adjusted covariates. Thus, we conducted this study to determine the sex-specific associations between various obesity indices (WC, WHR, and BMI) and albuminuria in the general Korean population. We used the Fifth Korea National Health and Nutrition Examination Survey (KNHANES V-2) data from 2011 and targeted non-diabetic and non-hypertensive healthy adults to minimize the influences of traditional risk factors for CKD.

## Methods

### Study population

This study was based on data acquired in the KNHANES V-2. The KNHANES has been conducted periodically since 1998 to assess the health and nutritional status in the civilian, non-institutionalized Korean population. The KNHANES V is a cross-sectional and nationally representative survey that was conducted by the Korea Centers for Disease Control and Prevention (KCDC) from 2010 to 2012. Urine albumin was first measured starting in 2011 (KNHANES V-2), and 3840 households were selected using a stratified, multistage probability sampling design. A total of 8518 individuals completed the health interview and health examination surveys. We included 6308 adults (age 20–79 years) and excluded subjects who took antihypertensive or antidiabetic medications, those who reported a history of chronic renal failure or cancer, females who were pregnant or menstruating during the health examination, and subjects with missing urine albumin data. Finally, 3841 participants (1730 males and 2111 females) were selected as our study population.

KNHANES was conducted in accordance with the Ethical Principles for Medical Research Involving Human Subjects. All of the participants provided informed consent prior to participation in the study. This study was approved by the Institutional Review Board of Hallym University Sacred Heart Hospital (IRB approval number 2013-I075).

### Data collection and measurements

Data were collected through standardized health examinations conducted in specially equipped mobile examination centers and via face-to-face interviews inside households. The health survey sequence involved intake, receipt of written informed consent, anthropometric measurements, blood sampling, and completion of a questionnaire survey. A standardized questionnaire regarding age, socioeconomic characteristics, medical history, drug use, smoking habit and other lifestyle risk factors was prepared.

Anthropometric data were measured according to standardized guidelines. Height and weight were obtained using standardized techniques and equipment. Height was measured to the nearest 0.1 cm using a portable standiometer (Seriter, Bismarck, ND, USA). Weight was measured to the nearest 0.1 kg using a Giant-150 N calibrated balance-beam scale (Hana, Seoul, Korea). BMI was calculated by dividing weight by the square of height (kg/m^2^). WC was measured to the nearest 0.1 cm during exhalation, using a measuring tape (SECA 200, SECA, Chino, CA, USA) at the horizontal plane midway between the inferior costal margin and the iliac crest at the mid-axillary line. The WHR was calculated as WC in cm divided by height in cm. Systolic and diastolic blood pressure (BP) was measured by standard methods using a sphygmomanometer (Baumanometer; Baum, Copiague, NY, USA) with the patient in the sitting position after a 5-min rest period. Three measurements were obtained with a 30-s interval. The mean of the second and third measurements was used for analysis. Pulse pressure was calculated as the difference between systolic and diastolic BP. Blood samples were collected in the morning after at least an 8-h fast, and single-spot urine specimens were collected from the first morning void. Analysis of fasting plasma glucose and serum total cholesterol, triglyceride, low-density lipoprotein cholesterol and high-density lipoprotein cholesterol levels was performed with a Hitachi Automatic Analyzer 7600 (Hitachi, Tokyo, Japan). Smoking status was classified according to three categories of never smoker, former smoker, and current smoker. Alcohol consumption was categorized into three groups of never drinker, former drinker, and current drinker. Physical activity was defined as >150 min of moderate intensity activity or 60 min of vigorous intensity activity per week.

### Obesity indices and albuminuria

We divided the subjects into four groups based on sex-specific quartiles (I–IV) of the obesity indices (BMI, WC, and WHR). The quartile criteria for males and females were as follows. For males, BMI: 14.4 ≤ I < 21.8 kg/m^2^, 21.8 ≤ II < 23.9 kg/m^2^, 23.9 ≤ III < 26.0 kg/m^2^, 26.0 ≤ IV < 45.1 kg/m^2^; WC: 52 ≤ I < 77 cm, 77 ≤ II < 84 cm, 84 ≤ III < 90 cm, 90 ≤ IV < 137 cm; and WHR: 0.32 ≤ I < 0.46, 0.46 ≤ II < 0.50, 0.50 ≤ III < 0.53, 0.53 ≤ IV < 0.78. For females, BMI: 13.2 ≤ I < 20.6 kg/m^2^, 20.6 ≤ II < 22.6 kg/m^2^, 22.6 ≤ III < 25.1 kg/m^2^, 25.1 ≤ IV < 42.2 kg/m^2^; WC: 55 ≤ I < 70 cm, 70 ≤ II < 76 cm, 76 ≤ III < 83 cm, 83 ≤ IV < 116 cm; and WHR: 0.34 ≤ I < 0.45, 0.45 ≤ II < 0.49, 0.49 ≤ III < 0.54, 0.54 ≤ IV < 0.75. Urine albumin and creatinine concentrations were measured in random urine samples using a turbidimetric immunoassay and colorimetric method, respectively (Hitachi Automatic Analyzer 7600). The ratio of urinary albumin to urinary creatinine is reported as the albumin–creatinine ratio (uACR) in mg/g creatinine. Albuminuria was defined as uACR ≥30 mg/g Cr [17].

### Statistical analysis

The weighted mean and standard error (SE), or proportion (%) and SE of the general characteristics according to hypertension status were calculated considering the complex sampling design of the KNHANES. Continuous data are expressed as means and SEs, and categorical data as frequencies and SEs, as appropriate. We used Student’s *t* test to compare continuous variables and the Chi-squared test to compare categorical variables between groups. The triglyceride and uACR values were log transformed to improve the normality of the distributions. The uACR was assigned a value of 1 mg/g in cases in which the urine albumin level was below the detection threshold to allow for log transformation. A multivariate logistic regression was performed to estimate the adjusted odds ratio (OR) and 95 % confidence interval (CI) and assess the risk for albuminuria according to the obesity indices. We adjusted for age, lifestyle (smoking and physical activity), high BP, and impaired fasting glucose and triglyceride levels in the logistic regression analyses. All tests were two-sided, and *p* < 0.05 was considered to indicate significance. All analyses were performed using PASW Statistics 21.0 (SPSS Inc., Chicago, IL, USA).

## Results

### General study subject characteristics according to the presence of albuminuria

The sex-specific general characteristics of the study subjects according to the presence of albuminuria are presented in Table 1. Mean age, BMI, WC, WHR, BP, glucose, glycated hemoglobin, and triglyceride levels were significantly higher in the albuminuria group than those in the normoalbuminuria group in both sexes. A higher proportion of female smokers was in the albuminuria group. The prevalence of albuminuria was 3.5 % in the total study population (4.2 % in females and 2.9 % in males).

**Table 1:** Baseline characteristics of the study subjects according to the presence of albuminuria

	Males (*n* = 1730)			Females (*n* = 2111)		
	Normoalbuminuria	Albuminuria	*P*	Normoalbuminuria	Albuminuria	*P*
Age (years)	40.67 (0.48)	50.26 (2.07)	<0.001	42.76 (0.43)	49.2 (1.86)	0.001
Height (cm)	171.64 (0.23)	169.35 (0.72)	0.002	158.08 (0.18)	156 (0.86)	0.017
Weight (kg)	70.82 (0.39)	72.08 (2.27)	0.582	57.22 (0.28)	59.46 (1.16)	0.063
Body mass index (kg/m^2^)	23.99 (0.11)	25.04 (0.64)	0.108	22.90 (0.11)	24.35 (0.40)	0.001
Waist circumference (cm)	83.79 (0.35)	87.96 (1.85)	0.025	76.68 (0.27)	81.79 (1.18)	<0.001
Waist-to-height ratio	0.489 (0.002)	0.519 (0.01)	0.002	0.486 (0.002)	0.525 (0.008)	<0.001
Systolic BP (mmHg)	117.8 (0.44)	126.7 (2.20)	<0.001	111 (0.42)	129.3 (3.08)	<0.001
Diastolic BP (mmHg)	78.9 (0.34)	83.8 (1.47)	0.001	72.4 (0.27)	79.7 (1.46)	<0.001
Pulse pressure	38.9 (0.27)	42.8 (1.68)	0.02	38.7 (0.28)	49.6 (2.17)	<0.001
Hemoglobin A1c (%)	5.56 (0.02)	5.82 (0.10)	0.013	5.5 (0.01)	5.7 (0.08)	0.017
Fasting glucose (mmol/L)	94.4 (0.48)	103.2 (2.85)	0.003	90.9 (0.37)	96.6 (1.97)	0.005
Total cholesterol (mmol/L)	189.4 (1.33)	203.8 (4.52)	0.002	189.5 (0.95)	207.9 (4.08)	<0.001
Triglyceride (mmol/L)	151.6 (3.95)	200.3 (17.5)	0.001	103.7 (1.99)	144 (8.44)	<0.001
LDL-C (mmol/L)	115.1 (1.53)	115.9 (7.53)	0.918	111.8 (1.37)	128.7 (7.77)	0.032
HDL-C (mmol/L)	44.37 (0.72)	46.25 (5.91)	0.756	53.24 (0.74)	52.28 (4.25)	0.819
uACR (mg/g)	3.25 (0.14)	192.72 (62.67)	<0.001	3.75 (0.13)	146 (35.7)	<0.001
eGFR (ml/min/1.73 m^2^)	94.06 (0.54)	90.11 (1.94)	0.049	99.19 (0.60)	97.28 (2.45)	0.435
Smoker, *n* (%)						
Never	20.8 (1.2)	19.9 (5.8)	0.687	85.1 (1.0)	74.3 (5.3)	0.023
Former	29.3 (1.5)	36.9 (8.9)		6.4 (0.7)	7.4 (2.8)	
Current	47.8 (1.6)	42.1 (9.4)		6.5 (0.7)	15.7 (5.3)	
Alcohol, *n* (%)						
Never	3.8 (0.6)	6.5 (3.0)	0.435	11.6 (0.9)	15.6 (4.9)	0.510
Former	6.6 (0.8)	3.5 (2.0)		14.8 (1.0)	18 (5.1)	
Current	87.5 (1.0)	89 (4.1)		71.7 (1.2)	63.8 (6.4)	
Physical activity, *n* (%)						
Inactive	76.6 (1.3)	73.3 (7.0)	0.619	81.8 (1.1)	86.9 (3.5)	0.222
Active	23.4 (1.3)	26.7 (7.0)		18.2 (1.1)	13.1 (3.5)	

Data are mean ± standard error or an estimated percentage (standard error), as appropriate

*LDL-C* low-density lipoprotein cholesterol, *HDL-C* high-density lipoprotein cholesterol, *uACR* urinary albumin-to-creatinine ratio, *MDRD eGFR* modification of diet in renal disease estimated glomerular filtration rate

### Factors associated with albuminuria

Table 2 shows the ORs for albuminuria in the sex-specific quartiles for the obesity indices and other potential confounders from the univariate logistic regression. Increased age, BP, fasting glucose and triglyceride levels resulted in a higher risk for albuminuria in both sexes. Current smoking resulted in an increased risk for albuminuria in females only. The second quartile group of all obesity indices was used as the reference group, and the ORs for albuminuria in each quartile group were calculated. The quartile group of females for all obesity indices had an increased risk for albuminuria compared to those in the second quartile group. The ORs of the fourth quartile group for WC, WHR, and BMI were 2.79 (95 % CI 1.51–5.16), 2.54 (95 % CI 1.38–4.67), and 2.63 (95 % CI 1.39–4.97). However, the risk for albuminuria in males was not different between the reference group and the other groups.

**Table 2:** Factors associated with albuminuria in the univariate logistic regression

	Males		Females	
	OR	(95 % CI)	OR	(95 % CI)
Age (years)	1.05	(1.03–1.07)	1.03	(1.02–1.05)
Systolic BP (mmHg)	1.04	(1.02–1.06)	1.05	(1.04–1.07)
Diastolic BP (mmHg)	1.05	(1.02–1.07)	1.08	(1.05–1.10)
High blood pressure^a^	3.08	(1.65–5.74)	6.29	(3.45–11.37)
Impaired fasting glucose^b^	2.17	(1.15–4.07)	2.26	(1.24–4.13)
Triglyceride (mmol/L)				
<1.69	1		1	
1.69 to <2.26	1.46	(0.53–4.00)	2.98	(1.58–5.62)
2.26 to <5.65	3	(1.40–6.42)	2.61	(1.28–5.28)
≥5.65	1.34	(0.36–5.07)	9.56	(1.58–58)
Waist circumference (cm)				
I	0.59	(0.20–1.74)	0.75	(0.32–1.74)
II	1		1	
III	1.37	(0.65–2.89)	0.63	(0.26–1.53)
IV	1.68	(0.66–4.29)	2.79	(1.51–5.16)
Waist-to-height ratio				
I	0.51	(0.18–1.42)	0.48	(0.19–1.20)
II	1		1	
III	0.95	(0.40–2.27)	1.22	(0.57–2.60)
IV	1.94	(0.78–4.83)	2.54	(1.38–4.67)
Body mass index (kg/m^2^)				
I	0.55	(0.21–1.46)	0.89	(0.40–1.99)
II	1		1	
III	0.66	(0.28–1.52)	1.44	(0.67–3.09)
IV	1.41	(0.56–3.52)	2.63	(1.39–4.97)
Smoking				
Never	1		1	
Former	1.32	(0.56–3.09)	1.32	(0.58–3.00)
Current	0.92	(0.39–2.15)	2.77	(1.25–6.15)
Alcohol				
Never	1		1	
Former	0.31	(0.08–1.22)	0.9	(0.37–2.16)
Current	0.6	(0.21–1.75)	0.66	(0.32–1.38)
Physical activity				
Active	1		1	
Inactive	0.84	(0.41–1.69)	1.47	(0.79–2.73)

*CI* confidence interval, *OR* odds ratio

^a^High blood pressure was defined as systolic blood pressure ≥140 mmHg or diastolic blood pressure ≥90 mmHg

^b^Fasting blood glucose ≥5.55 mmol/L

### ORs for albuminuria across sex-specific quartiles of the obesity indices

Multivariate-adjusted ORs for albuminuria across sex-specific quartiles of the obesity indices are shown in Table 3. We adjusted for confounding variables, such as age, smoking status, and physical activity, in models 1 and 2. The ORs of the fourth quartile group of all obesity indices for females showed a significantly higher risk compared to those in the reference group. However, the risk for albuminuria in the fourth quartile group of WHR lost significance (OR 1.84; 95 % CI 0.94–3.63) in model 3, which was further adjusted for high BP and impaired fasting glucose and triglyceride levels, whereas the fourth quartile group of WC and BMI still showed a clear increased risk for albuminuria (OR 1.96; 95 % CI 1.03–3.74 and OR 2.24; 95 % CI 1.15–4.37). Males tended to show an increased risk of albuminuria according to their WC and WHR, but the associations did not reach statistical significance.

**Table 3:** Multivariate-adjusted odds ratios for albuminuria across sex-specific quartiles of the obesity indices

	Model 1^a^	Model 2^b^	Model 3^c^
	OR (95% CI)	OR (95% CI)	OR (95% CI)
Males			
WC			
I	0.63 (0.22–1.86)	0.52 (0.17–1.61)	0.60 (0.19–1.86)
II	1	1	1
III	1.28 (0.61–2.70)	1.25 (0.58–2.69)	1.03 (0.45–2.34)
IV	1.71 (0.66–4.43)	1.74 (0.67–4.57)	1.28 (0.44–3.78)
*P* for trend	0.045	0.028	0.306
WHR			
I	0.61 (0.21–1.72)	0.55 (0.18–1.65)	0.73 (0.24–2.22)
II	1	1	1
III	0.84 (0.35–2.01)	0.89 (0.36–2.16)	0.83 (0.33–2.05)
IV	1.70 (0.68–4.25)	1.81 (0.70–4.64)	1.43 (0.52–3.92)
*P* for trend	0.039	0.023	0.306
BMI			
I	0.50 (0.19–1.28)	0.44 (0.16–1.19)	0.51 (0.19–1.34)
II	1	1	1
III	0.60 (0.26–1.39)	0.60 (0.26–1.40)	0.48 (0.20–1.16)
IV	1.69 (0.65–4.41)	1.76 (0.65–4.78)	1.26 (0.40–3.97)
*P* for trend	0.058	0.043	0.366
Females			
WC			
I	0.88 (0.37–2.05)	0.79 (0.33–1.93)	0.77 (0.30–2.00)
II	1	1	1
III	0.58 (0.24–1.38)	0.60 (0.24–1.46)	0.50 (0.20–1.25)
IV	2.44 (1.30–4.58)	2.29 (1.17–4.49)	1.96 (1.03–3.74)
*P* for trend	0.015	0.015	0.018
WHR			
I	0.55 (0.22–1.37)	0.54 (0.21–1.36)	0.59 (0.23–1.56)
II	1	1	1
III	1.09 (0.51–2.35)	1.11 (0.51–2.41)	0.95 (0.43–2.10)
IV	2.19 (1.15–4.19)	2.12 (1.07–4.19)	1.84 (0.94–3.63)
*P* for trend	0.011	0.018	0.009
BMI			
I	1.07 (0.47–2.46)	0.93 (0.39–2.19)	0.99 (0.40–2.41)
II	1	1	1
III	1.32 (0.61–2.83)	1.24 (0.56–2.75)	1.15 (0.54–2.49)
IV	2.58 (1.36–4.90)	2.40 (1.24–4.68)	2.24 (1.15–4.37)
*P* for trend	0.003	0.005	0.019

*CI* confidence interval, *OR* odds ratio, *WC* waist circumference, *WHR* waist-to-height ratio, *BMI* body mass index

^a^Adjusted for age

^b^Adjusted for age, lifestyle (smoking and physical activity)

^c^Adjusted for age, lifestyle (smoking and physical activity), high blood pressure, and impaired fasting glucose and triglyceride levels

According to the subgroup analysis of the relationships between each obesity index and albuminuria according to the menopausal status of female subjects, we found that those in the fourth quartile of postmenopausal women had a significantly higher risk for albuminuria than did those in the second quartile group according to their WC and BMI (OR 2.47; 95 % CI 1.01–5.89 vs. OR 4.46; 95 % CI 1.66–11.95), whereas no such association was observed in premenopausal women (supplementary Table A).

## Discussion

In this large nationally representative study, we demonstrated an association between obesity and albuminuria in non-diabetic, non-hypertensive females, but not in males. Among various obesity indices in females, the highest quartile WC and BMI groups had a twofold higher risk of albuminuria than that in the reference group (second quartile group) after adjusting for several potentially confounding factors. Our cutoff points for the highest WC and BMI quartile to predict albuminuria were 83 cm and 25.1 kg/m^2^, respectively, which were similar to the obesity thresholds used in another Asian population [18]. Although none of the obesity indices was associated with albuminuria in Korean males, the risk for albuminuria tended to increase with WC and WHR, but not BMI.

Several studies have reported that obesity is a risk factor for the development and progression of CKD and albuminuria [19, 20]. Furthermore, systematic reviews suggest that weight loss intervention is associated with improved estimated glomerular filtration rate and albuminuria [21, 22]. Several possible mechanisms explain how obesity increases the risk for albuminuria. First, adipose tissue is a source of several active compounds, such as tumor necrosis factor-α, interleukin-6, monocyte chemoattractant protein-1, C-reactive protein, plasminogen activator inhibitor-1, resistin, and free fatty acids. These adipokines cause renal damage by inducing sympathetic overactivity, systemic inflammation, and oxidative stress [20]. Second, adipocytes secrete hormones, including adiponectin and leptin, which regulate podocyte function. Decreasing serum adiponectin and increasing serum leptin levels is a consistent feature among obese patients and may induce microalbuminuria [23]. Third, visceral obesity and associated insulin resistance lead to hyperinsulinemia and activate the renin-angiotensin system, which can lead to glomerular hypertension, endothelial dysfunction, vasoconstriction, and matrix proliferation and expansion [4, 24].

Sex-specific associations were an important part of this study. Previous studies have reported significant associations between obesity and CKD in males and females [7–9, 25]. However, several Asian studies have suggested sex-specific associations between obesity and renal dysfunction or albuminuria. Studies from Singapore and Japan show that higher BMI is associated with CKD and proteinuria only in males [10–12, 26]. In contrast, Chinese studies have reported that abdominal obesity is related to CKD and albuminuria only in females [13, 14], which was similar to our results.

In this study, we first demonstrated sex-specific associations between obesity and albuminuria in the general population. These associations were more prominent in females, especially those who were postmenopausal, but were not apparent in males. The reason for the insignificant association between obesity and albuminuria in males is unclear. Despite previous studies reporting that the male sex is associated with a more rapid rate of progression of renal disease [27], the prevalence of albuminuria in our study was higher in females (4.2 %) than that in males (2.9 %). Using the standardized uACR value of ≥30 mg/g Cr to define microalbuminuria may underestimate microalbuminuria in men, whereas it may overestimate it in women, because the muscle mass and urinary excretion of creatinine in women are usually lower than those in men [28].

One possible reason for the sex difference in the association between obesity and albuminuria is that sex hormones affect the kidneys and influence the progression of renal disease, including mesangial proliferation, matrix accumulation, activation of the rennin-angiotensin system, and synthesis and release of cytokines [29, 30]. 17β-Estradiol suppresses total collagen synthesis by mesangial cells, whereas testosterone does not affect collagen synthesis. In addition, estrogens may exert potent antioxidant actions in the mesangium, which slows the development of glomerulosclerosis and provide a protective effect against the progression of renal disease in females [31]. This finding suggests that sex could act as a renal disease modifier. Because we excluded subjects with self-reports of chronic renal disease, more females with slowly progressing renal disease may have been included in our study.

Another possible reason is that the impact of potential confounders on albuminuria may be greater than the strength of the association with obesity in males. The mechanism of how obesity affects renal damage by sex should be clarified in future studies. In addition, the abdominal obesity indices (WC and WHR), but not BMI, tended to increase the risk for albuminuria in males. Because BMI could not be stratified for muscle mass and fat mass, subjects with higher muscle mass may have been misclassified into the obese group.

Our study had several strengths. First, we used data from a large nationally representative sample, which could warrant generalizability. Second, we minimized the influences of traditional risk factors for CKD and focused on the associations between obesity and albuminuria with respect to sex. We selected a healthy population without hypertension or diabetes, stratified according to sex, and adjusted for a wide range of confounding factors. Despite these strengths, there were limitations to this study. First, this study had a cross-sectional design, which did not allow us to make causal inferences. Second, we did not collect urine samples or analyze 24-h urinary albumin excretion. Instead, we used a single uACR result, which may have misclassified some albuminuria cases. However, spot urine uACR is commonly used to monitor kidney damage and predict cardiovascular risk in clinical practice [32].

In this nationally representative study, we investigated sex-specific associations between various obesity indices (WC, WHR, and BMI) and albuminuria in the general Korean population. We demonstrated a significant association between obesity and albuminuria in females, but not in males. Among women, a higher WC or BMI was significantly associated with the risk for albuminuria. These results provide putative evidence that obesity should be considered an independent risk factor for albuminuria in women, and future studies should clarify the mechanisms underlying such sex differences.

## Electronic supplementary material

Below is the link to the electronic supplementary material.

Supplementary material 1 (DOCX 12 kb)

## Abbreviations

WC: Waist circumference
BMI: Body mass index
WHR: Waist-to-height ratio
uACR: Urine albumin–creatinine ratio
KNHANES: Korean National Health and Nutrition Examination Survey
