# Supplementary material for: Sex differences in the association between obesity and albuminuria among Korean adults: a cross-sectional study using the Korea National Health and Nutrition Examination Survey data
Source: Clin Exp Nephrol. 2016 Feb 22;21(1):27–34. doi: 10.1007/s10157-016-1238-z (PMC5283500; doi:10.1007/s10157-016-1238-z)
Supplement: Supplementary file 1 — Supplementary material 1 (DOCX 12 kb) [file 10157_2016_1238_MOESM1_ESM.docx]

**Table A**. Associations between obesity indices and albuminuria according to menopausal status

|  |  | Premenopausal  (*n* = 1212, 69.1%) | Postmenopausal  (*n* = 849, 30.9%) |
| --- | --- | --- | --- |
|  |  | OR^a^ (95% CI) | OR^a^ (95% CI) |
| Waist circumference | I | 0.84 (0.28–2.52) | 0.32 (0.06–1.61) |
|  | II | 1 | 1 |
|  | III | 0.16 (0.03–0.90) | 1.05 (0.32–3.47) |
|  | IV | 1.60 (0.58–4.42) | 2.47 (1.04–5.89) |
| Waist/hip ratio | I | 0.85 (0.27–2.66) | 0.01 (0.001–0.05) |
|  | II | 1 | 1 |
|  | III | 1.10 (0.36–3.37) | 0.75 (0.24–2.30) |
|  | IV | 1.67 (0.53–5.28) | 1.97 (0.80–4.89) |
| BMI | I | 0.87 (0.27–2.78) | 1.07 (0.34–3.38) |
|  | II | 1 | 1 |
|  | III | 0.66 (0.20–2.15) | 2.25 (0.81–6.29) |
|  | IV | 1.49 (0.54–4.11) | 4.46 (1.66–11.95) |

^a^ Adjusted for age, lifestyle (smoking and physical activity), high blood pressure, and impaired fasting glucose and triglyceride levels.
